# Focal epilepsy without overt epileptogenic lesions: no evidence of microstructural brain tissue damage in multi-parametric quantitative MRI

**DOI:** 10.3389/fneur.2023.1175971

**Published:** 2023-07-17

**Authors:** Celona Hamid, Michelle Maiworm, Marlies Wagner, Susanne Knake, Ulrike Nöth, Ralf Deichmann, René-Maxime Gracien, Alexander Seiler

**Affiliations:** ^1^Department of Neurology, Goethe University Hospital, Frankfurt, Germany; ^2^Brain Imaging Center, Goethe University Frankfurt, Frankfurt, Germany; ^3^Center for Personalized Translational Epilepsy Research (CePTER) Consortium, Frankfurt, Germany; ^4^Institute of Neuroradiology, Goethe University Hospital, Frankfurt, Germany; ^5^Epilepsy Center Hessen and Department of Neurology, Philipps-University Marburg, Marburg, Germany

**Keywords:** epilepsy, quantitative MRI, tissue microstructure, voxel-wise analyses, tissue segmentation, brain networks

## Abstract

**Background and purpose:**

In patients with epilepsies of structural origin, brain atrophy and pathological alterations of the tissue microstructure extending beyond the putative epileptogenic lesion have been reported. However, in patients without any evidence of epileptogenic lesions on diagnostic magnetic resonance imaging (MRI), impairment of the brain microstructure has been scarcely elucidated. Using multiparametric quantitative (q) magnetic resonance imaging MRI, we aimed to investigate diffuse impairment of the microstructural tissue integrity in MRI-negative focal epilepsy patients.

**Methods:**

27 MRI-negative patients with focal epilepsy (mean age 33.1 ± 14.2 years) and 27 matched healthy control subjects underwent multiparametric qMRI including T1, T2, and PD mapping at 3 T. After tissue segmentation based on synthetic anatomies, mean qMRI parameter values were extracted from the cerebral cortex, the white matter (WM) and the deep gray matter (GM) and compared between patients and control subjects. Apart from calculating mean values for the qMRI parameters across the respective compartments, voxel-wise analyses were performed for each tissue class.

**Results:**

There were no significant differences for mean values of quantitative T1, T2, and PD obtained from the cortex, the WM and the deep GM between the groups. Furthermore, the voxel-wise analyses did not reveal any clusters indicating significant differences between patients and control subjects for the qMRI parameters in the respective compartments.

**Conclusions:**

Based on the employed methodology, no indication for an impairment of the cerebral microstructural tissue integrity in MRI-negative patients with focal epilepsy was found in this study. Further research will be necessary to identify relevant factors and mechanisms contributing to microstructural brain tissue damage in various subgroups of patients with epilepsy.

## 1. Introduction

Epilepsy is defined as a chronic condition with a sustained predisposition to epileptic seizures and resulting neurobiological, cognitive, psychological and social consequence (1). Consistent with the concept of epilepsy as a condition potentially affecting the entire brain, pathological changes of the cerebral microstructural tissue integrity in brain areas which exceeded or were remote to the putative epileptogenic focus or the presumed seizure onset zone have been reported in studies employing structural magnetic resonance imaging (MRI) and diffusion tensor imaging (DTI) (2, 3). Those findings include atrophy of the cerebral gray matter (GM) and extensive microstructural damage to the cerebral white matter (WM) in patients with temporal lobe epilepsy (TLE), malformations of cortical development and primary generalized epilepsy (2–7).

Besides structural imaging and DTI, quantitative (q)MRI has been used to investigate potential microstructural alterations in epilepsy patients within brain tissue appearing normal on conventional MRI. In contrast to DTI, which allows for the assessment of microstructural tissue in terms of the integrity of microstructural boundaries via the measurement of water diffusion (2), qMRI provides aggregate parameters at the voxel level that reflect microscopic tissue properties in a more differentiated manner (8, 9). Therefore, qMRI techniques may provide more profound insights into various aspects of pathological tissue alterations by depicting several distinct microstructural processes. While the interpretation of conventional MRI is mainly based on image contrast, qMRI mapping is value-based. A previous study applying quantitative T2 mapping in patients with TLE found increased T2 values even in patients without evidence of tissue atrophy or overt conspicuities in image contrast on conventional MRI (10). Furthermore, in a more recent work on patients with focal cortical dysplasia (FCD), widespread increases of cortical T2 values, widely exceeding the brain region harboring the FCD, were observed (11). These findings indicated global microstructural alterations in the cortical gray matter (GM) (11), since T2 is known to be sensitive to abnormalities in the relative myelin content, tissue iron deposition, the extra- and intracellular water content and gliotic tissue conversion (8, 12–14). Findings of cerebral microstructural tissue abnormalities have been interpreted as mainly reflecting secondary brain tissue damage as the consequence of continuously repeated ictal activity and its detrimental effects on the microstructural tissue integrity via structural and functional brain networks and cortical interconnections (10).

In general, most of the studies which assessed pathological changes of the microstructural brain tissue integrity with quantitative or structural imaging techniques included patients with known epileptogenic lesions such as FCD (11), hippocampus sclerosis previously diagnosed by conventional MRI protocols (10) or mixed patient collectives with focal epilepsies due to epileptogenic structural abnormalities of various etiologies and genetic epilepsies (15, 16). In the respective patient cohorts, the probability of extended microstructural tissue damage might be relatively high due to the presence of an already present circumscribed and clearly localized epileptogenic pathology and seizure onset zone.

In this study, we sought to investigate whether epilepsy patients with inconspicuous structural MRI (MRI-negative) and without evidence suggesting a genetic or metabolic etiology, exhibit signs of a microstructural pathology as a correlate of altered functional brain networks in comparison to a cohort of age-matched healthy control subjects. For this purpose, multiparametric qMRI with T1, T2, and proton density (PD) mapping was used, together with tissue segmentation based on synthetic anatomies and comprehensive analyses of qMRI parameters, including both a region-of-interest (ROI)-based approach and voxel-wise analyses.

## 2. Materials and methods

### 2.1. Participants

A total of 27 epilepsy patients (mean age 33.1 ± 14.2 years) for whom neuroradiological assessment based on clinical 3 Tesla (T) MRI data including an epilepsy-specific protocol (17) did not unveil a structural lesion (MRI-negative) and 27 healthy control subjects (mean age 33.0 ± 13.8 years) were recruited. The sex distribution was equal in both groups (*n* = 12 (44.4%) females in the patient and in the control group, respectively). The study was approved by the local IRB (Ethik-Kommission des Fachbereichs Medizin der Goethe-Universität). The patients/participants provided written informed consent to participate in this study. The study was performed according to the principles formulated in the Declaration of Helsinki.

### 2.2. Acquisition of MRI data

A 3T MAGNETOM TRIO MR scanner (Siemens Healthineers, Erlangen, Germany) was used for MRI data acquisition. This scanner is equipped with a body coil required for radio frequency (RF) transmission and with a phased-array head coil with 8 channels for signal reception.

T2 mapping was based on four fast spin echo (SE) datasets acquired with different TE. The parameters of the acquisition were the same as described earlier (11): matrix size = 256 × 176, slice thickness = 2 mm (no inter-slice gap), spatial resolution = 1 × 1 × 2 mm^3^, number of axial slices = 69, interleaved slice sampling, field of view (FOV) = 256 × 176 mm^2^, TR = 10 s, TE = [13, 67, 93, 106] ms, turbo factor = 13, echo spacing = 13.3 ms, bandwidth = 176 Hz/pixel, parallel imaging (reduction factor of 2), partial Fourier factor 6/8, refocusing angle = 160°, acquisition time for each dataset 1:32 min. Because each of the datasets was acquired twice for averaging purposes, the total acquisition time was 12:16 min.

For B1 mapping, a reference gradient echo (GE) and a magnetization prepared GE dataset were acquired. An RF-pulse followed by a crusher gradient was utilized for magnetization preparation, rotating the longitudinal magnetization by an angle β (nominal value β_0_ = 45 °). The other acquisition parameters were: matrix size = 64 × 56, slice thickness = 4 mm (no gap), isotropic spatial resolution = 4 mm, number of sagittal slices = 40, FOV = 256 × 224 mm^2^, TR = 11 ms, TE = 5 ms, α = 11°, centric phase encoding, bandwidth=260 Hz/pixel, duration = 0:53 min.

B0 mapping was based on two GE datasets with different TE: matrix size, slice thickness, FOV, isotropic resolution, number of sagittal slices as described above for B1 mapping, TR = 560 ms, TE [1,2] = [4.89, 7.35] ms, bandwidth = 200 Hz/pixel, α = 60°, duration = 1:03 min, export of magnitude and phase data.

For T1 and PD mapping two spoiled GE datasets at different excitation angles (α_1, 2_) were recorded. This approach results in different signal intensities (I) in both images. The parameters were: same volume coverage as described for B0 and B1-mapping, matrix size = 256 × 224 × 160, FOV = 256 × 224 × 160 mm^3^, isotropic spatial resolution = 1 mm, TR =16.4 ms, TE = 6.7 ms, α_1, 2_ = [4, 24]°, bandwidth = 222 Hz/pixel, acquisition duration (for both datasets): 9:48 min.

Signal loss in the variable flip angle (VFA) data caused by T2^*^ relaxation effects occurring during the finite TE of 6.7 ms were compensated. To this aim, two GE datasets with different TE were recorded. The acquisition parameters were: same volume coverage as for T1, B1, and B0 mapping, matrix-size = 128 × 112, FOV = 256 × 224 mm^2^, isotropic spatial resolution = 2 mm, number of sagittal slices = 80, slice thickness = 2 mm (no gap), TR = 1,336 ms, TE [1,2] = [4.3, 11] ms, α = 50°, bandwidth = 292 Hz/pixel, acquisition duration for both datasets: 5 min.

### 2.3. Quantitative MRI (qMRI) mapping

The analyses were performed with custom-built Perl (version 5.30) and Matlab scripts. Functions and programs included in the FMRIB Software Library (FSL, Oxford, version 5.0.7) (18), in Matlab [MathWorks, Natick, MA, version R2012b (8.0.0.783)], and in FreeSurfer (Athinoula A. Martinos Center for Biomedical Imaging, Boston, version 6.0.1) (19) were used.

The single fast SE datasets for T2 mapping were co-registered to a common reference to account for motion artifacts. Subsequently, the datasets with identical TE were averaged for SNR improvement. T2 was estimated by exponentially fitting the dependence between the signal intensities in the averaged T2-weighted datasets and TE. Correction for the influence of stimulated echoes was performed as reported in the literature (20).

The applied B1 mapping algorithm was previously described in the literature (21). In summary, the magnetization prepared dataset was divided by the reference dataset (without magnetization preparation) to determine the cosine of the local preparation angle β B1 then followed from the quotient of β and the nominal value β_0_. For B0 mapping, the phase differences between the two GE datasets acquired with different TE were analyzed with FSL PRELUDE and FUGUE.

The measurement of T1 was based on the VFA method (22). To account for motion artifacts, the two datasets acquired at different excitation angles were co-registered. Subsequently, the excitation angles α_1, 2_ = [4, 24]° and the resulting signal intensities I_1, 2_ were utilized to plot I_i_/sin(α_i_) vs. I_i_/tan(α_i_), yielding a straight line with the slope exp(-TR/T1) from which preliminary T1 maps were derived and subsequently corrected for B1 and B0 inhomogeneities and for insufficient spoiling of the transverse magnetization (23).

Measurement of PD was performed as reported in the literature (24). In summary, the co-registered VFA dataset acquired with the lower excitation angle (which is PD weighted) was further processed to compensate for T2^*^, T1, and B1 effects and for the specific profile of the receive-coil.

For segmentation purposes, synthetic T1-weighted magnetization-prepared rapid acquisition of gradient echoes (MP-RAGE) datasets were calculated from quantitative T1 maps as described previously (25, 26). The following virtual acquisition parameters were utilized: matrix size = 256 × 224 × 160, field of view = 256 × 224 × 160 mm^3^, isotropic resolution = 1 mm, TR = 1,900 ms, TI = 900 ms, α = 9°, echo spacing = 8.1 ms.

### 2.4. Data post-processing and analysis

The tissue segmentation was performed via the “recon-all” command implemented in Freesurfer utilizing the synthetic MP-RAGE datasets. PD and T1 maps, which had the same orientation as the synthetic anatomies, were transferred to the Freesurfer space and the tool BBRegister (27) was used for the boandary-based coregistration of the T2 maps to the synthetic MP-RAGE anatomies.

#### 2.4.1. Cortical analysis

**Figure 1:**
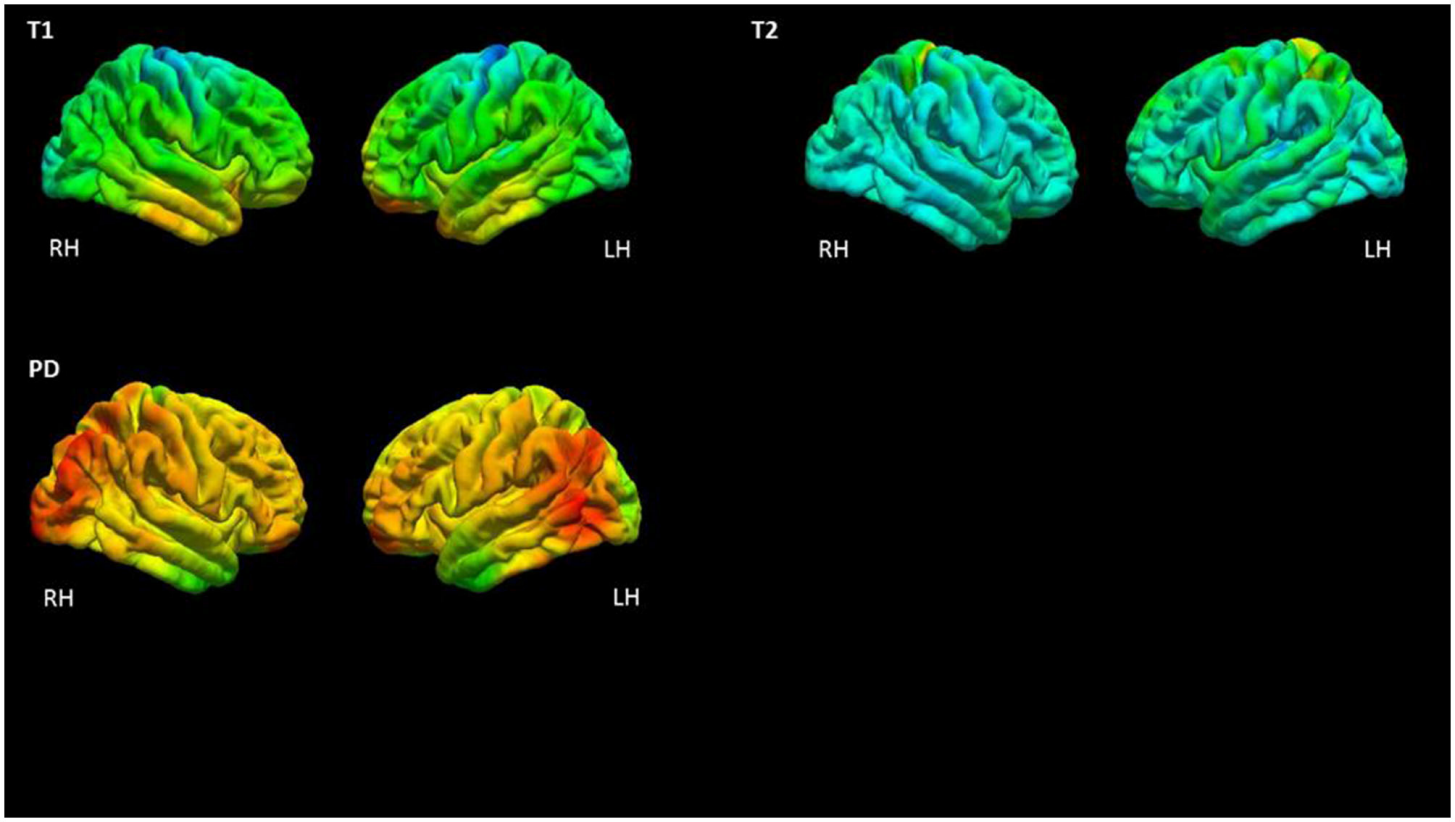
Illustration of qMRI values of a representative epilepsy patient after sampling from the middle 20% of the cortical ribbon and mapping to the surface (corresponding to a projection fraction 0.4–0.6 of the entire cortical layer). Values are shown on the pial surface of the average subject (“fsaverage”) created by the Freesurfer software after smoothing with a Gaussian kernel (full width at half maximum = 10 mm) as used for the GLM analysis. RH, right hemisphere; LH, left hemisphere; ms, milliseconds; p.u., percent units.

For analysis of the cortical GM, the T1, T2, and PD values were read in the middle 20% of the cortical layer (28) and stored in surface datasets. This approach was followed to reduce partial volume effects (PVE) with WM and cerebrospinal fluid (CSF). For the analysis of global qMRI parameter values across the entire cortex as an initial evaluation for group comparisons, average cortical T1, T2, and PD values were calculated for each subject, including all non-zero vertices into this calculation. Values were compared between both groups via two-sided unpaired *t-*tests. For surface-based cortical group analysis, the T1, T2, and PD surface datasets were normalized to the average subject (“fsaverage”) and smoothed (Gaussian kernel, full width at half maximum = 10 mm). Cortical T1, T2, and PD values after normalization and smoothing are shown for a representative epilepsy patient in Figure 1. Employing the script mri_glmfit, general linear model (GLM) analyses were calculated for surface-based comparisons between groups. Permutation simulations (vertex-wise threshold = 0.001, cluster-wise threshold = 0.05) were performed to identify clusters indicating significant differences between groups and to compensate for multiple comparisons.

#### 2.4.2. Region-of-interest-based WM and deep GM analysis

WM masks excluding WM lesions and combined bilateral deep GM masks (including the caudate nucleus, putamen, thalamus and pallidum) were derived from the Freesurfer segmentation results. Partial volume effects from cerebrospinal fluid (CSF) were reduced by eliminating pixels with T1 values > 2,000 ms from the masks (29). The masks were co-registered to the T2 maps, while the T1 and PD maps had the same orientation as the MP-RAGE datasets and the resulting tissue masks. Similar to the analyses performed in cortical GM, mean parameter values were extracted from the WM and deep GM ROIs as a first evaluation and compared between groups using two-sided unpaired *t*-tests.

#### 2.4.3. Voxel-wise WM and deep GM analysis

**Figure 2:**
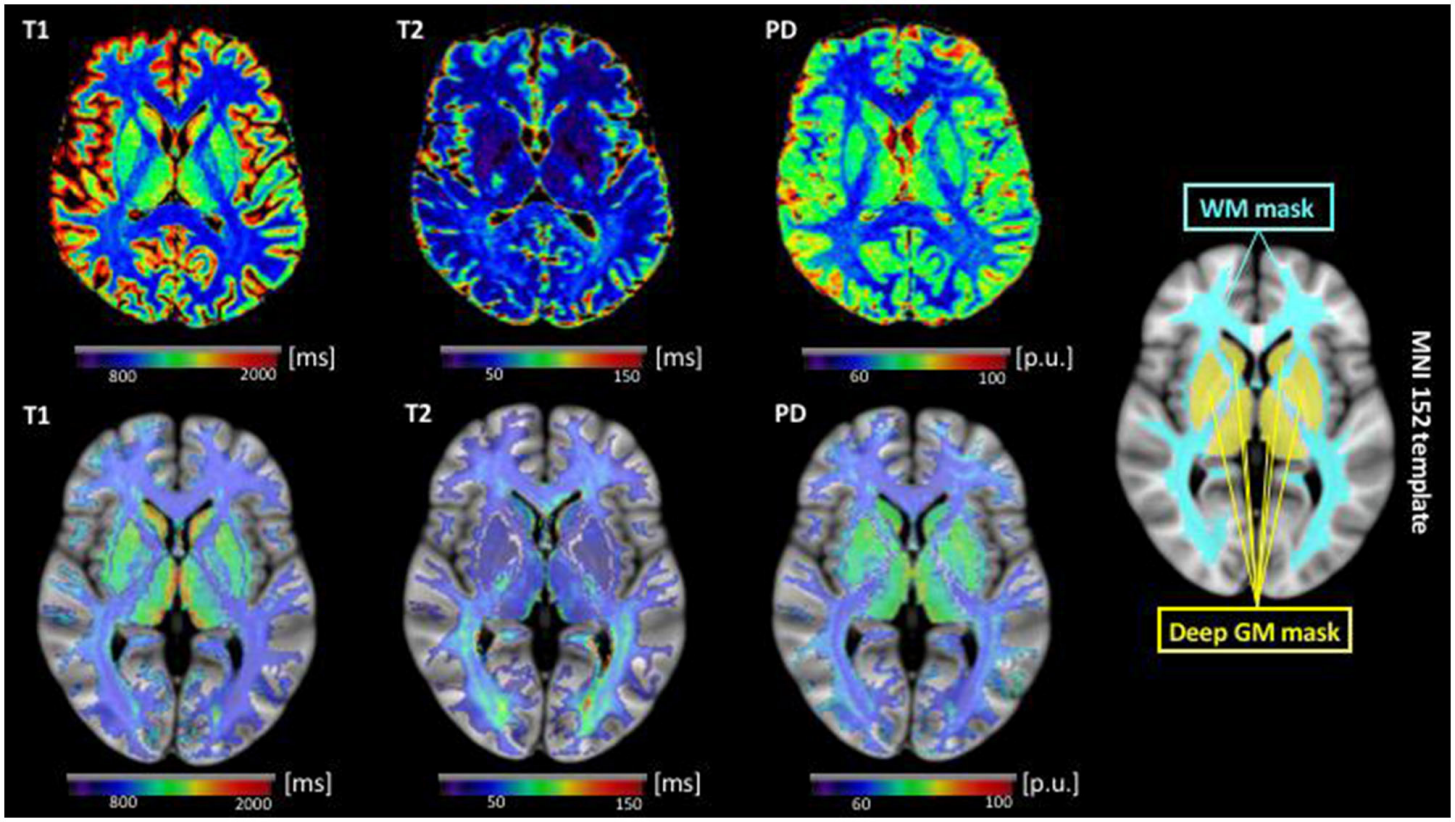
Illustration of qMRI parametric maps after co-registration to the respective synthetic anatomy **(top row)** and data preparation for the voxel-wise analysis of the cerebral WM and deep GM in MNI standard space **(bottom row)** for a representative epilepsy patient (same as in Figure 1). In the **bottom** row, below each parametric map, the corresponding WM and deep GM extracted from the parametric map by the means of the individual tissue segmentation is shown after non-linear co-registration to MNI standard space. Here, a transparency of 50% on the background was applied to the parametric maps to allow for visualization of the alignment of the segmented maps with the underlying anatomy. For the voxel-wise analysis between patients and control subjects with FSL randomize, the calculations were restricted to the binary WM and deep GM masks shown on the right, for minimization of partial volume effects. ms, milliseconds; p.u., percent units; WM, white matter; GM, gray matter; MNI, Montreal Neurological Institute.

First, the quantitative T2 datasets were linearly co-registered to the synthetic anatomies using FSL FLIRT. For the PD and T1 maps, this step was not required since these parameter maps had already the same orientation as the anatomies. The WM masks excluding WM lesions and the deep GM masks obtained from the Freesurfer segmentation were applied to the quantitative maps to isolate voxels in these regions in order to generate T1, T2 and PD maps of the WM and the deep GM. Spatial normalization of the MP-RAGE anatomies into Montreal Neurological Institute (MNI) 152 space was performed by non-linear registration (FSL FNIRT) after linear initialization with FLIRT (Figure 2). Afterwards, the co-registration matrices (from the T2 map to the MP-RAGE anatomies for the T2 values and from the MP-RAGE datasets to MNI space) for all parameters were applied to the WM and deep GM T1, T2 and PD maps for normalization (Figure 2). Voxel-wise statistical group comparisons were calculated with RANDOMIZE as included in the FSL toolbox, using threshold-free cluster enhancement (TFCE) to compensate for multiple comparisons. The cluster-wise significance level was set to *p* < 0.05.

### 2.5. Statistical analysis

Two-sided unpaired *t*-tests were used to perform group comparisons of the qMRI mean parameter values in cortical GM, deep GM, and cerebral WM. *P*-values below 0.05 were considered significant for all statistical tests. Due to the exploratory character of the study and the small number of tests with three parameters investigated, no correction for multiple comparisons was performed for the group comparisons of mean parameter values in the cortical and deep GM as well as in the cerebral WM.

## 3. Results

### 3.1. Clinical baseline characteristics of epilepsy patients

In the majority of patients, the epileptogenic zone was located either in the frontal (*n* = 9) or in the temporal lobe (*n* = 11), according to recurrent localization-typical or stereotyped seizures and focal interictal or ictal epileptiform discharges on electroencephalography (EEG). The median number of antiseizure medication (ASM) was *n* = 2 in the patient group [interquartile range (IQR) 1–3]. The median number of seizures in the last 3 months before inclusion in the study was 4 (IQR 0–12). *N* = 8 patients (29.6%) had been completely seizure-free in the last 3 months before enrolment. Demographic and clinical baseline characteristics for patients and healthy control subjects are summarized in Table 1.

**Table 1:** Demographic and clinical baseline characteristics for patients and control subjects.

	**Patients (*n =* 27)**	**Control subjects (*n =* 27)**
Age [years] (Mean ± SD)	33.1 ± 14.2	33.0 ± 13.8
Female sex [*n* (%)]	12 (44.4%)	12 (44.4%)
Disease duration [years] (Mean ± SD)	12.2 ± 7.72	
Seizures in the last 3 months [n] [Median (IQR)]	4 (0–12)	
Present ASM [n] [Median (IQR)]	2 (1–3)	
Overall ASM in medical history [n] [Median (IQR)]	3 (2–6)	
**Epileptogenic zone [*****n*** **(%)]**		
A frontal/frontocentral	9 (33.3%)	
B temporal	11 (37.1%)	
C occipital/temporooccipital	4 (14.8%)	
D other/unknown^a^	4 (14.8%)	

SD, standard deviation; IQR, interquartile range; ASM, antiseizure medication. ^a^localization of epileptogenic zone not exactly known (suspected left-hemispheric: *n* = 2, suspected right-hemispheric: *n* = 1, completely unknown: *n* = 1).

### 3.2. Analysis of qMRI parameters

#### 3.2.1. Cortical qMRI parameters

**Figure 3:**
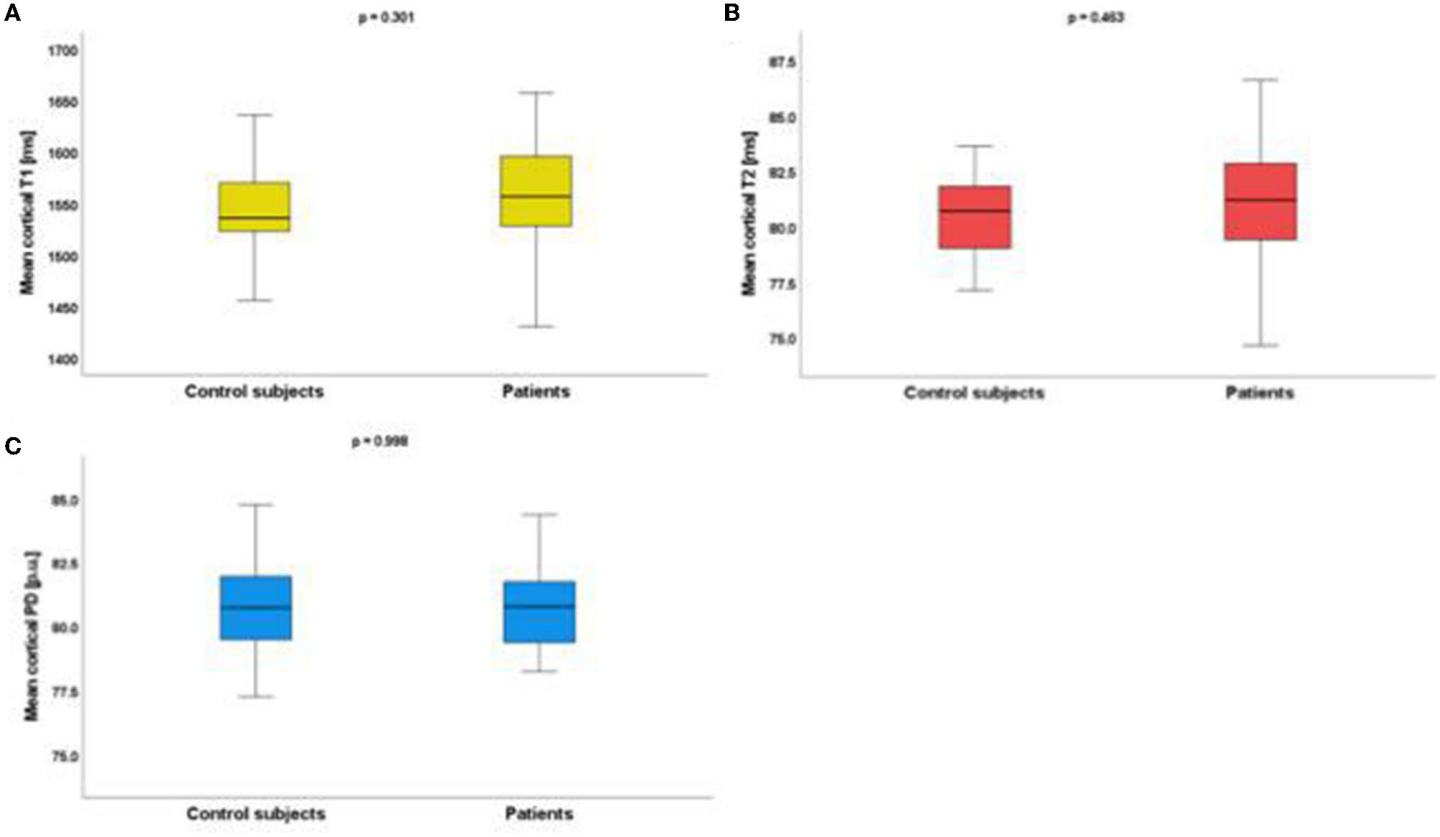
Boxplots illustrating mean values of cortical T1 **(A)**, T2 **(B)**, and PD **(C)** (calculated by averaging across all vertices) for patients and control subjects. No significant differences for the analyzed qMRI parameters were found between the groups. ms, milliseconds; p.u., percent units.

After averaging of the cortical qMRI parameter values across the surface, there were no significant differences for mean cortical T1 (patients vs. controls: 1,556.79 ± 51.14 vs. 1,543.58 ± 41.28 ms), T2 (patients vs. controls: 80.97 ± 2.82 vs. 80.46 ± 2.21 ms), and PD values (patients vs. controls: 80.74 ± 2.00 vs. 80.74 ± 1.74 p.u.) between patients and control subjects (Figure 3). Furthermore, the surface-based cortical GLM-analysis did not reveal any cluster indicating group differences after compensation for multiple comparisons, neither for T1, T2 nor for PD.

#### 3.2.2. QMRI parameters in the cerebral WM and deep GM

The ROI-based analyses of qMRI parameters for cerebral WM and deep GM did not reveal any significant differences for mean T1, T2, and PD values between the groups. Mean values and SD for T1, T2, and PD in cerebral WM and deep GM are summarized in Table 2. Furthermore, the voxel-wise analysis performed in MNI standard space did not show any significant cluster indicating significant differences for the investigated qMRI parameters on a group level.

**Table 2:** Mean qMRI parameter values obtained from the cerebral white matter and deep gray matter in patients and control subjects.

	**Patients (*n =* 27)**	**Control subjects (*n =* 27)**	***p*-value**
***WM***			
T1 [ms] (Mean ± SD)	894.07 ± 57.08	886.48 ± 57.1	0.298
T2 [ms] (Mean ± SD)	62.15 ± 12.99	62.66 ± 12.59	0.431
PD [p.u.] (Mean ± SD)	66.24 ± 3.41	66.46 ± 3.47	0.789
***Deep GM***			
T1 [ms] (Mean ± SD)	1,249.95 ± 158.94	1,242.18 ± 157.37	0.287
T2 [ms] (Mean ± SD)	58.6 ± 12.47	60.33 ± 13.21	0.762
PD [p.u.] (Mean ± SD)	75.79 ± 5.93	76.24 ± 5.56	0.993

WM, white matter; GM, gray matter; ms, milliseconds; SD, standard deviation; p.u., percent units.

## 4. Discussion

In this study, multiparametric qMRI was used to investigate possible pathological alterations of the brain tissue microstructure in patients with focal epilepsies and inconspicuous structural MR imaging at a group level. To this end, mean qMRI parameter values across the entire cortical GM, the deep GM, and the cerebral WM were analyzed along with voxel-wise group comparisons of qMRI parameter values for these tissue compartments in standard space. Voxel-based quantification (VBQ) (30) is an alternative approach of performing tissue-specific analyses of qMRI parameter maps, which in principle is similar to the approach employed in this study and has been well-described in the literature. We found no significant differences between the patient and the control group for global qMRI parameter values across the different tissue classes. Furthermore, no significant clusters for group differences in qMRI parameters could be identified in voxel-based analysis.

Since increased network connectivity with hyperexcitability and neuronal hypersynchronization must be hypothesized in epilepsy patients with a sustained predisposition to epileptic seizures, it is generally plausible to assume that patients with focal epilepsy may exhibit pathological alterations of the cerebral tissue microstructure despite unremarkable conventional MRI without evidence of epileptogenic lesions. However, given the lack of significant differences in qMRI parameters between patients and healthy control subjects, our results do not point toward a relevant impairment of the cerebral microstructural tissue integrity in epilepsy patients without overt epileptogenic lesions in structural imaging. The qMRI parameters acquired in this study cover a broad range of microstructural processes potentially involved in pathological tissue remodeling. PD is a surrogate marker of microstructural tissue atrophy, since increased PD values indicate an enlargement of the interstitial space and thus a (relative) reduction of the local tissue volume fraction (31, 32). Quantitative T1 mapping is sensitive to changes of the tissue water and myelin content as well as tissue iron deposition (33, 34), while T2 mapping especially detects demyelination, microstructural axonal injury and gliotic tissue conversion (13, 14). Furthermore, quantitative T2 is particularly sensitive to tissue net water uptake, including both an enlargement of the extracellular space due to interstitial edema (e.g., due to increased permeability of the blood-brain-barrier) and cellular swelling due to intracellular edema, e.g. caused by excitotoxicity (35–37).

Based on structural imaging with volumetric assessment of the cerebral GM, pathological changes of the cerebral microstructure reflected by tissue atrophy and progressive cortical thinning have been described in a variety of epilepsy syndromes (15, 16, 38). Several of these studies included mixed patient collectives, which were not explicitely stratified according to the presence of a putative epileptogenic lesion (15, 16, 38). Patients with TLE represented the majority of patients, respectively an important subgroup in those studies (15, 16, 38). Since seizure activity is potentially based on or even contributes to altered structural networks, an association between seizure frequency and atrophic tissue alterations seems generally plausible. However, studies investigating the correlation between seizure frequency and cerebral atrophy yielded inconsistent results and the association could not be shown in a reproducible manner (16, 39–41). For instance, a comprehensive longitudinal study by Liu et al. found no association between structural parameters and seizure recurrence (16). Rather, an association between atrophy and exposure to multiple anticonvulsive drugs, driven by neurotoxic side effects of the antiepileptic medication, was suggested (16). Galovic et al. did not find any association between accelerated cortical thinning and seizure frequency or the antiepileptic drug load (38).

The microstructural processes underlying or potentially preceding cerebral atrophy in epilepsy patients are largely unknown. A recent study on epilepsy patients with FCD but no evidence of cortical atrophy reported widespread prolongation of cortical T2 relaxation times, with increases of cortical T2-values extending far beyond the area harboring the FCD, both in the ipsilateral and the contralateral hemisphere (11). The mechanisms underlying the observed cortical T2 changes are not entirely clear. Since a previous study described that hippocampal T2 increases correlate with gliosis in patients with TLE (42), it is conceivable that gliotic tissue changes either cause a prolongation of the tissue parameter T2 or influence the T2 measurement (11). Cortical regions harboring the FCDs were mainly located in the frontal and temporal lobe as well as in the cingulum, where also the most pronounced cortical T2 increases were detected on a group level after correction for multiple comparisons (11). FCDs were shown to alter the cerebral functional and structural connectivity (43, 44), a finding suggesting that FCDs might be the underlying cause of a network disease affecting the whole-brain network. Therefore, it might be possible that pathological microstructural tissue remodeling in patients with focal epileptogenic brain lesions is related to a pathological network reorganization with a sustainably altered neuronal activity, irrespective of the actual seizure frequency or the antiepileptic medication. Alterations of the large-scale brain network structure involving network hyperactivity and increased connectivity have been linked to network hyperexcitability, neuronal hypersynchronization and seizure predisposition (45–47), which are associated with (reactive) astrogliosis (48, 49). Furthermore, altered neuronal activity may yield an impact on the permeability of the blood-brain-barrier (50, 51) as another microstructural process affecting cortical T2.

Epilepsies result from a pathological alteration of a functional network instead of a specific region. Such alterations can be assumed to affect also MRI-negative epilepsy patients (52) but it can be speculated that they might be less pronounced with smaller and rather short-term effects on the functional connectivity and neuronal activity than for patients with abnormalities on structural imaging, thus potentially causing less widespread alterations of the tissue microstructure with a smaller magnitude. Therefore, in contrast to previous findings in patients with epileptogenic lesions, multiparamteric qMRI might not be sensitive enough to detect alterations of the cerebral microstructure in focal epilepsy patients with inconspicuous conventional MRI.

### 4.1. Limitations

Despite several strengths, which include especially the comprehensive qMRI protocol and the detailed analyses of various tissue classes, this study is not without limitations. First, the sample size in this study is relatively small, which might have limited the detectability of smaller changes in qMRI parameters in patients compared to controls. Furthermore, the epilepsy patients included in this study were heterogeneous in terms of the type of epilepsy, respectively the (presumed) seizure onset zone, disease duration, seizure frequency and antiepileptic medication (Table 1). We cannot exclude an impact of the heterogeneity concerning the clinical characteristics within the patient collective on our results. Since this is not a longitudinal study, we are not able to comment on potential changes of qMRI parameters in epilepsy patients across time and their relation to seizure frequency and ASM. Finally, although the applied qMRI techniques cover a variety of distinct microstructural processes in terms of their sensitivity to pathological tissue alterations, we cannot exclude that cerebral microstructural changes in epilepsy patients might not be detected with the methodology employed in this study. In order to investigate the nature of potential microstructural tissue alterations in patients with focal epilepsy, histological examinations along with a validation of qMRI parameters, e.g., in patients undergoing epilepsy surgery, would be of major interest.

## 5. Conclusions

This multiparametric qMRI study did not reveal any evidence of a relevant global or regional impairment of the brain microstructural integrity in focal epilepsy patients with inconspicuous structural imaging in terms of a potentially epileptogenic structural pathology, suggesting no permanent microstructural tissue damage related to seizure activity in these patients. A future study on a more homogeneous patient collective, which takes global and regional network structures and connectivity into account and allows for mapping of connectivity changes and network hyperexcitability, would be of interest to further evaluate the utility and sensitivity of multiparametric qMRI for detecting alterations of the cerebral microstructure in MRI-negative patients with focal epilepsy.

## Data availability statement

The original contributions presented in the study are included in the article/supplementary material, further inquiries can be directed to the corresponding author.

## Ethics statement

The studies involving human participants were reviewed and approved by Ethikkommission des Fachbereichs Medizin, Goethe-Universität Frankfurt. The patients/participants provided their written informed consent to participate in this study.

## Author contributions

CH: conceptualization of the study, literature research, collection of clinical data, data analysis and interpretation, and writing. MM: conceptualization of the study, literature research, collection of clinical and imaging data, data interpretation, and writing. MW and SK: conceptualization of the study, literature research, collection of clinical and imaging data, data interpretation, and critical review of the manuscript. UN and RD: development of the quantitative MR imaging technique, data analysis, and critical review of the manuscript. R-MG: conceptualization of the study, literature research, MR image analysis, and statistical. AS: conceptualization of the study, literature research, MR image analysis, statistical analysis, and writing. All authors contributed to the article and approved the submitted version.
